# Pharmacological Activation of the Bile Acid Nuclear Farnesoid X Receptor Is Feasible in Patients with Quiescent Crohn's Colitis

**DOI:** 10.1371/journal.pone.0049706

**Published:** 2012-11-26

**Authors:** Fiona D. M. van Schaik, Raffaella M. Gadaleta, Frank G. Schaap, Saskia W. C. van Mil, Peter D. Siersema, Bas Oldenburg, Karel J. van Erpecum

**Affiliations:** 1 Department of Gastroenterology and Hepatology, University Medical Center Utrecht, Utrecht, The Netherlands; 2 Department of Metabolic Diseases, University Medical Center Utrecht, Utrecht, The Netherlands; 3 Tytgat Institute for Liver and Intestinal Research, Academic Medical Center, Amsterdam, The Netherlands; Nihon University School of Medicine, Japan

## Abstract

**Background:**

The bile acid-activated nuclear receptor Farnesoid X Receptor (FXR) is critical in maintaining intestinal barrier integrity and preventing bacterial overgrowth. Patients with Crohn's colitis (CC) exhibit reduced ileal FXR target gene expression. FXR agonists have been shown to ameliorate inflammation in murine colitis models. We here explore the feasibility of pharmacological FXR activation in CC.

**Methods:**

Nine patients with quiescent CC and 12 disease controls were treated with the FXR ligand chenodeoxycholic acid (CDCA; 15 mg/kg/day) for 8 days. Ileal FXR activation was assessed in the fasting state during 6 hrs after the first CDCA dose and on day 8, by quantification of serum levels of fibroblast growth factor (FGF) 19. Since FGF19 induces gallbladder (GB) refilling in murine models, we also determined concurrent GB volumes by ultrasound. On day 8 ileal and cecal biopsies were obtained and FXR target gene expression was determined.

**Results:**

At baseline, FGF19 levels were not different between CC and disease controls. After the first CDCA dose, there were progressive increases of FGF19 levels and GB volumes during the next 6 hours in CC patients and disease controls (FGF19: 576 resp. 537% of basal; GB volumes: 190 resp. 178% of basal) without differences between both groups, and a further increase at day 8. In comparison with a separate untreated control group, CDCA affected FXR target gene expression in both CC and disease controls, without differences between both groups.

**Conclusions:**

Pharmacological activation of FXR is feasible in patients with CC. These data provide a rationale to explore the anti-inflammatory properties of pharmacological activation of FXR in these patients.

**Trial Registration:**

TrialRegister.nl NTR2009

## Introduction

The bile acid nuclear Farnesoid X Receptor (FXR) is the master regulator of bile acid homeostasis. FXR is mainly expressed in the ileum and liver, and regulates various genes encoding for bile acid transport proteins, including apical sodium-dependent bile acid transporter (ASBT) and ileal bile acid binding protein (IBABP) [1], [2]. Expression of the enterokine fibroblast growth factor (FGF)15 (human orthologue FGF19), which induces gallbladder (GB) refilling in the mouse, is also controlled by FXR [3]. It has been hypothesized that FGF15 functions as an “ileal brake” by signaling the end of the postprandial and return to the interdigestive phase. More recent data indicate a role for FXR in the regulation of lipid and glucose metabolism [4], [5].

There is clear evidence that the ileum is a key location where prevention of excessive intestinal inflammation and maintenance of intestinal barrier (both at the level of the small intestine and the colon) are orchestrated. Patients with Crohn's colitis (CC) are known to have an impaired antibacterial defense and impaired intestinal barrier function. For example, endogenous antimicrobial peptides such as α-defensins are produced in the ileum, and their levels are reduced in Crohn's disease, thereby compromising mucosal host defence [6]. In addition, phospholipid concentration and composition in the colonic mucus layer (pivotal in intestinal barrier function) are dependent on bile acid-induced phospholipid secretion in the ileum with subsequent spread to the distal colon by propulsory motility, and these are deficient in patients with Inflammatory Bowel Disease (IBD) [7], [8]. Finally, FXR has been implicated in maintaining intestinal barrier integrity and in the prevention of intestinal bacterial overgrowth [9]. According to recent data, patients with CC have an altered FXR expression in areas of inflamed mucosa [10]. In two murine models for colitis, we recently showed that the administration of a semi-synthetic FXR agonist ameliorates intestinal inflammation, with improvement of colitis symptoms, preservation of intestinal barrier function, reduced goblet cell loss and inhibition of proinflammatory cytokine expression [11]. The underlying mechanism for these anti-inflammatory effects is thought to be inhibition of NF-κB [11], [12]. Furthermore, we recently found reduced FXR target gene expression in the ileum of patients with clinically quiescent CC [13].

The aim of this study was to investigate whether pharmacological activation of FXR with its endogenous ligand chenodeoxycholic acid (CDCA) is feasible in patients with CC. As a read-out for FXR activation as well as to obtain more insight in the regulation of gallbladder motility in the fasted state, we also measured serum FGF19 levels and determined GB volumes after CDCA ingestion.

## Patients and Methods

### Ethics statement

This study was approved by the Institutional Ethics Committee of the University Medical Center Utrecht, the Netherlands, and the Central Committee on Research involving Human Subjects, the Hague, the Netherlands. Each patient gave written informed consent. The study was monitored by an independent external monitor. The study was registered at the Dutch Trial Register under number NTR2009 (www.trialregister.nl).

### Patients and protocol

Patients with clinically quiescent CC (Harvey-Bradshaw Index (HBI) ≤4) [14] and an indication for surveillance colonoscopy and disease controls who underwent colonoscopy for other clinical reasons were included if they consented in absence of exclusion criteria. Disease controls were excluded in case of previous inflammation of the gastrointestinal tract, with the exception of prior infectious gastroenteritis more than 6 months before the study. Additional exclusion criteria for both groups were: stool frequency >4/day; Body Mass Index >30 kg/m^2^ (potential interference with ultrasonographic GB volume measurements); C-reactive protein (CRP) >20 mg/L within three months before the study, aspartate transaminase (AST), alanine transaminase (ALT), lactate dehydrogenase (LDH), gamma glutamyl transpeptidase (GGT) or alkaline phosphatase (ALP) above upper limit of normal within 3 months before the study, abnormal prothrombin time (PT) or activated partial thromboplastin time (APTT); prior surgery of the gastro-intestinal tract (except appendectomy); previous cholecystectomy or papillotomy; GB or bile duct stones; concomitant primary sclerosing cholangitis or other significant hepatic or biliary pathology; any malignancy within 5 years before the study; use of steroids, cyclosporine, methotrexate, anti-TNF compounds, antibiotics, loperamide or codeine, laxatives or other drugs potentially interfering with CDCA (*e.g.* ursodeoxycholic acid or bile acid sequestrants) within one month before the study, and pregnancy or lactation. Four patients (three controls, one CC) were included in the study despite minimal increases of Alkaline Phosphatase (AF), gamma-glutamyltransferase (GGT) and APTT (resp. 2 U increase of AF in one patient, 2 U increase of GGT in another patient and 1 second increase of APTT in two patients), since these increases were thought not to lead to any safety concerns for these patients. Two included CC patients used oral anticoagulants and therefore APTT and PT were artificially increased during screening.

For activation of FXR we used CDCA (15 mg/kg body weight; Tramedico, Weesp, the Netherlands; Sigma-tau, Dusseldorf, Germany), which is the most potent endogenous FXR ligand in man. In contrast, the hydrophilic bile salt ursodeoxycholic acid (often used to treat cholestatic liver disease) has no effects on FXR activation. To get an impression of the extent of FXR activation in the enterocyte during CDCA, we also included for comparison a small separate control group of patients who had colonoscopy but no CDCA pretreatment. These controls were consecutively recruited at the outpatient clinic within the same time period as the CDCA-treated groups. Mean age of this control group was 61 years (±SD 5.9). Three controls were of female gender, one of male gender. Seven days before the colonoscopy, body weight, length and Harvey-Bradshaw index were assessed in the morning after an overnight fast. Next, baseline fasting GB volume was determined by ultrasound and blood was collected from a peripheral venous cannula for determination of plasma FGF19 level. After ingestion of CDCA, GB volume was determined and blood was sampled every hour during 6 hrs. The next six days CDCA (15 mg/kg) was ingested at bedtime. In case patients experienced side effects of CDCA ingestion, CDCA dosages were reduced with 50%. Dosages were again increased with one capsule (*i.e.* 250 mg) per day to the original dosage upon disappearance of side effects. Patients collected stools for 24 hrs at day 7. Patients fasted the night before colonoscopy (day 8), and ingested the last dose of CDCA in the early morning, before colonoscopy. Upon arrival at the outpatient clinic, compliance was measured by pill counts. Thereafter, fasting GB volume was determined and blood was collected. After bowel preparation (4L Colofort, Ipsen Farmaceutica, Hoofddorp, The Netherlands), patients underwent colonoscopy in the fasting state, during which biopsies were taken for histological evaluation and analysis of gene expression. For the latter, biopsies obtained from the ileum (n = 3) and caecum (n = 3) were immediately placed in liquid nitrogen and stored at −80°C until RNA isolation. All ileal biopsies were obtained in the distal ileum, immediately proximal to the ileal Bauhini valve. The protocol for this trial and supporting CONSORT checklist are available as supporting information; see Checklist S1 and Protocol S1.

### FGF19 analysis

FGF19 levels were assessed by a sandwich enzyme-linked immunosorbent assay specific for FGF19 as described elsewhere [15]. The following characteristics of FGF19 dynamics were assessed: baseline fasting FGF19 level prior to first CDCA ingestion (*FGF19_t0_*); minimal (*FGF19_min_*) and maximal (*FGF19_max_*) FGF19 level expressed in ng/mL and as percentage of *FGF19_t0_*, defined as the minimal resp. maximal FGF19 level during the 6 hrs after the first CDCA ingestion; time (in hrs) to reach *FGF19_min_* resp. *FGF19_max_* (*Time FGF19_min_*, *Time FGF19_max_*); maximal decrease resp. increase in FGF19 level compared to *FGF19_t0_* (*ΔFGF19_min_*_,_ *ΔFGF19_max_*) in ng/mL and as percentage of *FGF19_t0,_* and the area under the curve (AUC) of the changes in FGF19 level during 360 minutes after the first CDCA ingestion (in ng/mL*360 min.) calculated by the trapezoidal rule [16] as AUC  = 30*((X0+aX360)+2*(aX60+aX120+aX180+aX240+aX300)), in which X is the FGF19 level at the specific time point and ‘a’ is the change in FGF19 level compared to the fasting status (t = 0). Likewise, AUCs were calculated as percentages change from baseline (percent*360 min.). In addition, after 8 days of CDCA ingestion we assessed fasting FGF19 level (FGF19_t8) in ng/mL and as percentage of *FGF19_t0_,* and the increase in FGF19 level compared to *FGF19_t0_ (ΔFGF19_t8*) in ng/mL and as percentage of *FGF19_t0_*.

### Gallbladder volume measurement

GB volumes were determined by ultrasonography (5.0 MHz transducer, SDR 1500; Philips Ultrasound Inc., Santa Ana, CA, USA) after an overnight fast. Sagittal and transverse scans were obtained of the GB at its largest dimensions. GB volume was calculated with the sum-of-cylinders method [17]. For each time point, the mean of 3 measurements (at 5 min. intervals) was calculated. Parameters for evaluation of GB dynamics were similar to those assessed for FGF19, as described above.

### Fecal bile acid excretion

Bile acids were extracted from faeces with methanol/HCl (95:5 vol/vol) and quantified using an enzymatic assay (Diazyme Laboratories, Poway, USA).

### mRNA extraction and qRT-PCR analysis

Biopsies were homogenized (Omni TH tissue homogenizer, Omni International, Kennesaw, USA) and RNA was isolated using RNeasy Micro kit (Qiagen GmbH, Hilden, Germany) according to the manufacturer's instructions. The quantity, quality and integrity of isolated mRNA were determined by absorption measurement and RNA gel electrophoresis. Subsequently, cDNA was generated from 500 ng of total RNA using SuperScript II Reverse Transcriptase (Invitrogen, Carlsbad, CA, USA) and random hexamers (Roche, Basel, Switzerland). qRT-PCR analysis was carried out using SYBR green PCR master mix (Biorad, Veenendaal, The Netherlands) and a MyIQ real time PCR cycler (Biorad). Values were quantified using the comparative threshold cycle method. Transcript levels were normalized to hypoxanthine-guanine phosphoribosyltransferase (HPRT). Primers are listed in Table S1. Transcript levels were compared with a separate group of controls without inflammatory bowel disease who had a colonoscopy but no CDCA ingestion. The fold change in expression levels compared to these controls was assessed for CC and disease control groups on CDCA.

### Endpoints

The primary study endpoint was the difference between patients with CC and disease controls in change of fasting FGF19 level after 8 days of CDCA ingestion compared to baseline. Secondary study endpoints included differences between patients with CC and disease controls in: 1. change of fasting plasma FGF19 level after ingestion of the first dose of CDCA; 2. change of fasting GB volumes after ingestion of the first dose of CDCA; 3. expression of FXR and FXR target genes in ileal and caecal biopsies and 4. fecal bile acid excretion.

### Sample size calculation

In patients with CC, no data are available regarding the effect of CDCA on plasma FGF19 level, GB volumes or expression of FXR and FXR target genes in the enterocyte. Two previous studies reported mean fasting plasma FGF19 levels of 0.19–0.29 ng/mL in healthy individuals [15]; [18]. One of these studies demonstrated an increase of 250% after ingestion of CDCA in gallstone patients [18]. Assuming that the CDCA-stimulated increase of plasma FGF19 in gallstone patients would equal the increase in our disease controls and that the increase would be reduced with 30% in our patients with CC, 12 patients per group would be required to yield a statistical power of 0.8 (α = 0.05). We decided to end the study with slightly less CC patients (n = 9), because we were not able to recruit more patients despite extensive efforts.

### Statistical analysis

The effects of CDCA ingestion on GB volumes and FGF 19 levels during the first 6 hrs and potential differences between the two patient groups were compared by repeated measures ANOVA with Bonferroni correction. Differences between individual time points within each group were assessed by a paired t-test. Baseline characteristics and various individual parameters for GB dynamics, FGF19 dynamics and faecal bile acid excretion were compared between the two groups (patients with CC and disease controls) using Mann-Whitney U tests. For analysis of gene expression, fold-change relative to a group of untreated disease controls was compared between CDCA-treated CC patients and disease controls using a Mann-Whitney U test. Correlations between FGF19 levels and GB volumes or fecal bile acid excretion were explored by Spearman correlation. A two-sided P-value <0.05 was considered statistically significant. Statistical analysis was performed using SPSS 15.0.

## Results

### Clinical patient characteristics

**Figure 1:**
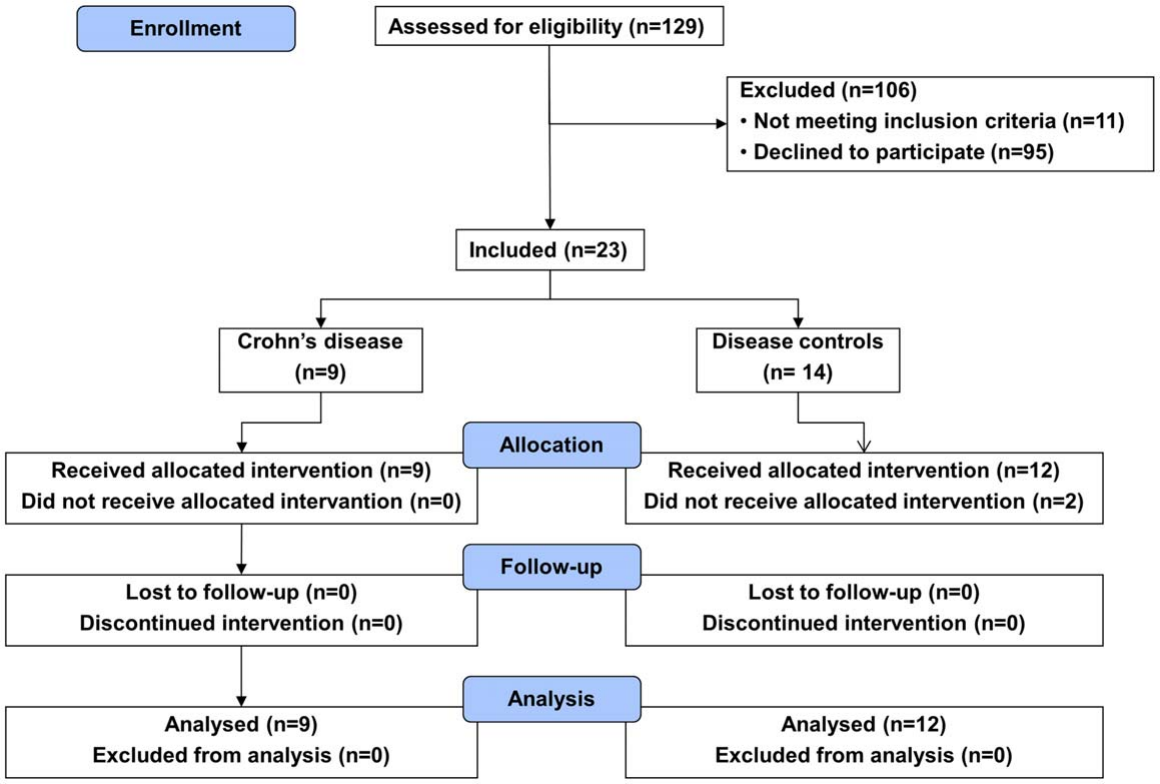
Flowchart of patient inclusion in the study.

Between September, 2009 and May, 2011 a total of nine patients with quiescent CC and 14 disease controls were enrolled in this study. Inclusion was not consecutive since only 23 of the potential 129 candidates consented to follow the demanding protocol and met the inclusion criteria (Figure 1). Two disease controls were included but did not receive CDCA, since baseline gallbladder volumes could not be reliably assessed with ultrasound. These patients were excluded from further analysis. Mean age was 51 years (±SD 15) in CC patients and 50 years (±SD 12) in disease controls. Six CC patients (67%) and six disease controls (50%) were of male gender. In the CC group, mean duration of disease was 20 years (SD ±10). Mean weight and BMI were respectively 75 kilograms (±SD 11) and 23.6 (±SD 2.2) in CC patients and 79 kilograms (±SD 15) and 24.8 (±SD 2.7) in disease controls. None of these characteristics were significantly different between the two groups. Disease characteristics of CC patients during previous and current colonoscopies are given in Table 1. In one patient from each patient group, the cecum was not reached and biopsies could therefore not be taken. In the short study period compliance with CDCA ingestion (based on pill counts at day 8) was 100% in all patients (taking in account dose reductions performed by the physician because of perceived side effects), except one who had by accident been taking one pill less for one day. Mean cumulative CDCA dose was 111 mg/kg * 8days (±SD 13.4) in CC patients and 117 mg/kg * 8 days (±SD 9.90) in disease controls (p = 0.26). Three CC patients and six disease controls experienced side effects of CDCA. These consisted of loose stools and bowel complaints in two CC patients and in five disease controls, and heartburn in one CC patient and one disease control. In one CC patient and five disease controls, dose reductions were needed because of these side effects. Mean duration of the reduced CDCA treatment period was 2.3 days (±SD 1.5). Results regarding FGF19 levels, gallbladder motility and fecal bile salt excretion in the two subgroups without CDCA dose reduction did not differ from the total groups (data not shown). There were no serious adverse events.

**Table 1:** Disease characte.ristics of patients with Crohn's colitis at previous investigations and current investigation.

	Patients with Crohn's colitis (N = 9)	
	Prior investigations	Current investigation
*Endoscopic disease extent*		
No inflammation	0 (0)	7 (78)
<50% of colon	6 (67)	1 (11)
>50% of colon	3 (33)	0 (0)
Unknown	0 (0)	1 (11)
*Histological disease extent*		
No inflammation	0 (0)	6 (67)
<50% of colon	2 (22)	2 (22)
>50% of colon	6 (67)	0 (0)
Unknown	1 (11)	1 (11)
*Endoscopic disease severity*		
No inflammation		7 (78)
Mild	3 (33)	2 (22)
Moderate	2 (22)	0 (0)
Severe	4 (44)	0 (0)
*Histological disease severity*		
No inflammation	0 (0)	7 (78)
Mild	3 (33)	0 (0)
Moderate	3 (33)	2 (22)
Severe	3 (33)	0 (0)
*Ileal involvement*	3 (33)	1 (11)
Unknown	0 (0)	1 (11)
*Disease behaviour* *		
Non-stricturing, non-penetrating (B1)	9 (100)	8 (89)
Stricturing (B2)	0 (0)	1 (11)
+ peri-anal disease	3 (33)	0 (0)

Data presented as numbers (% of group).

Disease extent was divided in more or less than 50% colonic involvement; disease extent of more than 50% was defined as involvement of three or more anatomical parts of the colon;

* disease behavior according to the Montreal Classification [24].

### Plasma FGF19 levels

**Figure 2:**
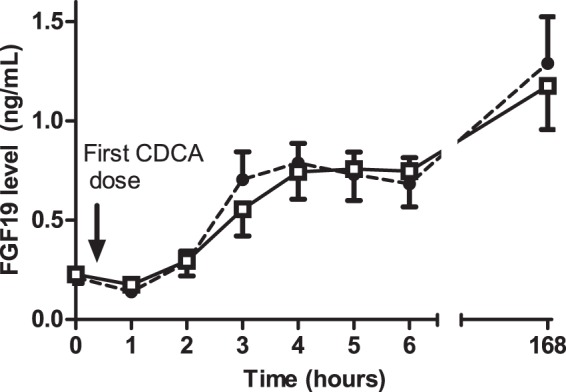
Plasma FGF19 levels (SEM) as a function of time after ingestion of chenodeoxycholic acid in patients with Crohn's colitis and disease controls. FGF19 levels progressively rise in all patients (p = 0.00) without differences between both groups 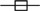 Crohn's patients; 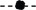 Disease controls.

At baseline, fasting plasma FGF19 levels were not different between CC and disease controls (0.23±0.14 resp. 0.21±0.11 ng/mL, mean ± SD; Table 2). One hour after the first CDCA dosage, mean FGF19 levels decreased to 0.18 ng/mL in CC patients (±SD 0.07, p = 0.24 compared to baseline) and to 0.14 ng/mL in disease controls (±SD 0.06, p = 0.006 compared to baseline). Thereafter, FGF19 levels progressively increased in all patients (p = 0.00, Figure 2) to an average of 576% and 537% of baseline levels in patients with CC and disease controls, respectively. No differences in FGF19 dynamics were found between CC patients and disease controls during the first 6 hours after CDCA ingestion (Table 2, Figure 2). After 8 days of CDCA ingestion FGF19 levels further increased to 1.18 ng/mL in CC patients (613% of baseline, p = 0.002) and 1.29 ng/mL in disease controls (626% of baseline, p = 0.000), again without differences between both groups.

**Table 2:** FGF19 dynamics in patients with Crohn's colitis and disease controls during the first 6 hours after CDCA ingestion and after 8 days of CDCA ingestion.

	Crohn's patients	Disease controls	
	N = 9	N = 12	p-value
***During the first 6 hrs after CDCA ingestion***			
*FGF19_t0* (ng/mL)	0.23 (0.14)	0.21 (0.11)	1.00
*FGF19_min_* (ng/mL)	0.14 (0.05)	0.13 (0.05)	0.48
*FGF19_min_* (%)	70 (28)	66 (23)	1.00
Δ*FGF19_min_* (ng/mL)	−0.09 (0.10)	−0.09 (0.08)	0.78
Δ*FGF19_min_* (%)	−30 (28)	−31 (23)	0.89
*Time FGF19_min_* (hours)	1.33 (1.32)	1.33 (0.89)	0.82
*FGF19_max_* (ng/mL)	1.00 (0.41)	0.96 (0.41)	0.48
*FGF19_max_* (%)	576 (330)	537 (292)	0.72
Δ*FGF19_max_* (ng/mL)	0.77 (0.46)	0.75 (0.37)	0.89
Δ*FGF19_max_* (%)	476 (330)	437 (292)	0.72
*Time FGF19_max_* (hours)	4.89 (1.05)	4.42 (1.24)	0.38
*AUC* (ng/mL*360min)	99.1 (107)	110 (81.0)	0.72
*AUC* (percent*360 min)	61352 (64680)	67291 (58258)	0.57
***After 8 days of CDCA ingestion***			
*FGF19_t8* (ng/mL)	1.18 (0.66)	1.29 (0.88)	0.89
*FGF19_t8* (%)	613 (301)	626 (193)	0.83
Δ*FGF19_t8* (ng/mL)	0.95 (0.64)	1.08 (0.72)	0.72
Δ*FGF19_t8* (%)	513 (301)	526 (193)	0.83

Values are in means ± SD; CDCA, chenodeoxycholic acid; *FGF19_t0,* baseline fasting FGF19 level; *FGF19_min_* (ng/mL), minimal FGF19 level in ng/mL; *FGF19_min_* (%), minimal FGF19 level as percentage of FGF_t0; *ΔFGF19_min_* (ng/mL), difference between FGFmin and FGF_t0; *ΔFGF19_min_* (%), percentage difference between FGFmin and FGF_t0; *Time FGF19_min_* (hours), time to minimal FGF19 level from t0; *FGF19_max_* (ng/mL), maximal FGF19 level; *FGF19_max_* (%), maximal FGF19 level as percentage of FGF_t0; *ΔFGF19_max_* (ng/mL), difference between FGFmax and FGF_t0; *ΔFGF19_max_* (%), percentual difference between FGFmax and FGF_t0; *Time FGF19_max_* (hours), time to maximal FGF19 level from t0; *AUC* (ng/mL*360 min), area under the curve of change of FGF19 level during 360 minutes; *AUC* (percent*360 min), area under the curve of percentage change of FGF19 level during 360 minutes; *FGF19_t8* (ng/mL), FGF19 level after 8 days of CDCA ingestion; *FGF19_t8* (%) FGF_t8 as percentage of FGF_t0; *ΔFGF19_t8* (ng/mL), difference between FGF19_t8 and FGF19_t0; *ΔFGF19_t8* (%), percentage difference between FGF19_t8 and FGF19_t0.

### Gallbladder motility

**Figure 3:**
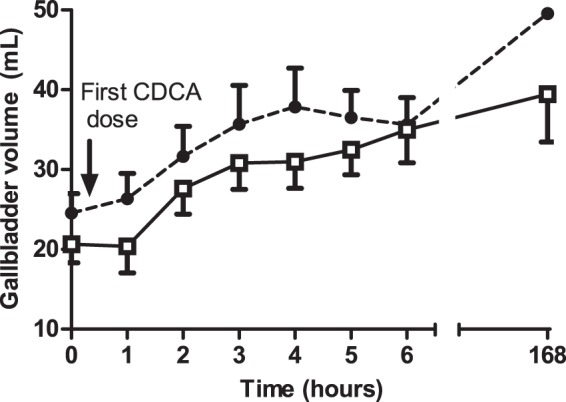
Gallbladder volume as a function of time after ingestion of chenodeoxycholic acid in patients with Crohn's colitis and disease controls. Gallbladder volumes progressively rise in all patients (p = 0.00) without differences between both groups 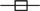 Crohn's patients; 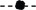 Disease controls.

GB dynamics after the first CDCA dosage are given in Table 3. Fasting, minimal and maximal GB volumes were slightly lower in patients with CC, although these differences did not reach statistical significance. In contrast to FGF19 dynamics, there was no initial decrease of GB volumes after the first CDCA dosage. Over time, a progressive increase in GB volume was seen in all CC patients and all but one disease controls (p = 0.00, Figure 3) to an average of 190% and 178% of baseline GB volumes in CC patients and disease controls, respectively. No differences in GB dynamics were found between CC patients and disease controls during the first 6 hours after CDCA ingestion, with the exception of time until maximum GB volume (*Time V_max_*), which was shorter in disease controls (Table 3, Figure 3). GB volumes further increased both in CC patients (199% of baseline, p = 0.004) and disease controls (208% of baseline, p = 0.000) at day 8.

**Table 3:** Gallbladder dynamics in patients with Crohn's colitis and disease controls during the first 6 hours after CDCA ingestion and after 8 days of CDCA ingestion.

	Crohn's patients	Disease controls	
	N = 9	N = 12	p-value
***During the first 6 hrs after CDCA ingestion***			
*V0* (mL)	20.7 (7.02)	24.6 (8.48)	0.29
*V_min_* (mL)	17.5 (7.17)	20.9 (7.11)	0.48
*V_min_* (%)	86 (18)	89 (20)	0.59
*ΔV_min_* (mL)	−3.1 (3.7)	−3.7 (6.4)	0.88
*ΔV_min_* (%)	−15 (18)	−12 (20)	0.59
*Time V_min_* (hours)	0.56 (0.53)	0.75 (1.42)	0.69
*V_max_* (mL)	36.3 (11.1)	41.4 (15.1)	0.36
*V_max_* (%)	190 (69)	178 (51)	0.83
*ΔV_max_* (mL)	15.7 (10.1)	16.9 (12.9)	1.00
*ΔV_max_* (%)	90 (68)	78 (51)	0.83
*Time V_max_* (hours)	5.11 (1.05)	3.67 (1.83)	0.04
*AUC* (mL*360 min)	2766 (2407)	3053 (3907)	0.89
*AUC* (percent*360 min)	16204 (15101)	15199 (15489)	0.94
***After 8 days of CDCA ingestion***			
*V_t8* (mL)	39.5 (18.0)	49.6 (20.7)	0.23
*V_t8* (%)	199 (79)	208 (94)	1.00
*ΔV_t8* (mL)	18.8 (14.3)	25.0 (17.4)	0.32
*ΔV_t8* (%)	99 (79)	108 (94)	1.00

Values are in means ± SD; CDCA, chenodeoxycholic acid; *V0*, baseline fasting gallbladder volume (mean of 3 measurements);*V_min_* (mL), minimal gallbladder volume; *V_min_* (%), minimal gallbladder volume as percentage of V0; *ΔV_min_* (mL), difference between Vmin and V0; *ΔV_min_* (%), percentual difference between Vmin and V0; *Time V_min_* (hours), time to minimal gallbladder volume from t0; *V_max_* (mL), maximal gallbladder volume; *V_max_* (%), maximal gallbladder volume as percentage of V0; *ΔV_max_* (mL), difference between Vmax and V0; *ΔV_max_* (%), percentual difference between Vmax and V0; *Time V_max_* (hours), time to maximal gallbladder volume from t0; *AUC* (mL*360 min), area under the curve of change of gallbladder volume during 360 minutes; *AUC* (percent*360 min), area under the curve of percentual change of gallbladder volume during 360 minutes; *V_t8* (mL), gallbladder volume after 8 days of CDCA ingestion; *V_t8* (%), *V_t8* as percentage of V0; *ΔV_t8* (mL), difference between V_t8 and V0; *ΔV_t8* (%), percentage difference between V_t8 and V0.

### Correlations between plasma FGF19 levels, gallbladder volumes and CDCA doses

Although a significant increase in both GB volume and FGF19 level over the first 6 hrs after CDCA ingestion was found (Figures 2 and 3), there was no significant correlation between plasma FGF19 levels and corresponding GB volumes at the individual time points after the first CDCA ingestion, neither in the two subgroups nor in the total group (R varying from −0.38 to 0.52, p>0.05).

### Faecal bile acid excretion

In one patient with CC, faecal bile acid excretion could not be assessed due to the absence of bowel movement on the day before the colonoscopy. Mean 24 hrs-faecal bile acid excretion after 7 days of CDCA ingestion was 2.23 mmol/24 hrs (± SD 2.54) in CC patients and 1.86 mmol/24 hrs (± SD 1.42) in disease controls (p = 0.68). In 7 of 8 CC patients and in 10 of 12 disease controls fecal bile acid excretion exceeded normal reference values (0.0−0.4 mmol/24 hrs). Fecal bile acid excretion did not correlate with cumulative ingested CDCA dose (data not shown). No significant correlations between FGF19 levels or GB volumes after 8 days of CDCA ingestion and fecal bile acid excretion were found (data not shown).

### FXR target gene expression

Transcript levels of FXR and FXR target genes for the two CDCA-treated groups are given in Table 4. Ileal expression of all investigated genes was not significantly different between both CDCA-treated groups. Compared to the separate untreated disease control group, in the (combined) CDCA-treated groups mRNA expression levels of the FXR target genes IBABP (3.40 versus 0.95, p = 0.00) and FGF19 (30.0 versus 0.87, p = 0.00) were significantly higher, whereas mRNA levels of ASBT were lower (0.48 versus 0.89, p = 0.03). Regarding FXR-dependent genes implicated in antibacterial defense: angiogenin 1 mRNA expression was significantly lower in the CDCA treated group (0.63 versus 0.88, p = 0.03). mRNA expression levels of FXR, FXR target and FXR dependent genes in cecum was much lower than in ileum, without differences between CDCA-treated CC and controls (data not shown).

**Table 4:** Ileal mRNA expression levels of FXR and FXR target genes in patients with Crohn's colitis and disease controls after CDCA stimulation.

	Crohn's patients	Disease controls	
	N = 8	N = 11	p-value
FXR	0.48 [0.31–1.43]	0.61 [0.40–0.81]	0.39
SHP	2.32 [0.48–8.52]	1.04 [0.37–4.57]	0.56
IBABP	3.15 [1.85–5.54]	4.15 [2.31–7.66]	0.30
FGF19	21.7 [3.37–862)	42.8 [2.98–909]	0.87
iNOS	0.80 [0.09–18.8]	1.03 [0.36–4.10]	0.74
Angiogenin 1	0.63 [0.28–0.98]	0.63 [0.36–0.94]	0.56
ASBT	0.46 [0.15–1.14]	0.58 [0.17–1.06]	0.74

Data in median [range]; p-values according to Mann-Whitney-U test.

Expression levels are given as fold change compared to a separate group of disease controls without CDCA stimulation (see text).

## Discussion

Since we previously observed dysregulation of FXR target gene expression in ileal biopsies of CC patients [13], in this study we aimed to explore whether pharmacological activation of ileal FXR is feasible in patients with CC. The main finding of our study is that activation of the bile acid-FXR-FGF 19 axis by the FXR ligand CDCA is feasible in patients with CC. The primary endpoint (increase of FGF19 levels after 8 days of CDCA ingestion) was not different between the two groups (0.95 ng/mL in CC patients resp. 1.08 ng/mL in disease controls). We included slightly less CC patients than originally intended, because we could not recruit more patients willing to undergo the demanding protocol. Nevertheless, it seems highly unlikely that different results regarding the primary endpoint would have been found with 12 CC patients. A post-sensitivity analysis revealed that inclusion of three more hypothetical CC patients assumed to have the lowest increase in plasma FGF19 level of the CC patient group would not have changed our results. Although we cannot exclude a difference in FXR activation between CD patients and controls in this study, our findings strongly direct to an adequate FXR activation in CC patients.

In order to get an impression of the extent of FXR activation in the enterocyte, we present mRNA levels as fold change from a separate control group not treated with CDCA. We found no difference in mRNA expression of FXR target genes in the ileal biopsies between the two CDCA-treated groups (Table 4). Although this lack of significant difference may have resulted from a small sample size, the increases in both FGF19 levels, which function as read-out of FXR-activation, and target gene expression, support the appropriate activation of the bile acid-FXR-FGF19 axis in the CC group. CDCA treatment increased ileal IBABP and FGF19 expression while reducing expression of ASBT. These changes are all expected upon FXR activation and appropriate in homeostasis of bile acid enterohepatic circulation to avoid potentially toxic intracellular bile acid concentrations. It should be noted in this respect that expression of FXR target genes, rather than FXR itself serve as indicators for FXR activation [19].

In the current study, no data are available on expression of FXR and target genes in the ileum of CC patients and disease controls before CDCA administration, since performing a second colonoscopy was considered not appropriate. Therefore, no conclusions can be made on FXR activity in the basal state. However, we previously reported that, under unstimulated conditions, there is lower expression of ileal FXR target genes in patients with CC [13] This could be a secondary event due to a disturbed enterohepatic circulation of bile salts: absorption of bile acids in the ileum into the enterohepatic circulation may be impaired in inflammatory bowel disease, either through active ileal inflammation or through faster passage of intestinal contents through the intestinal tract [20], [21]. In addition, lower FXR target gene expression in CC patients under unstimulated conditions could be secondary to minor intestinal inflammation, considering the existence of reciprocal inhibition of FXR and various genes encoding pro-inflammatory cytokines [10]; [19]. Finally, decreased FXR activation in CC patients could hypothetically be the result of overrepresentation of loss of function variants of the FXR gene in this patient category [22]. Our current results argue against the latter possibility, which is in line with our recent study in which we genotyped seven common tagging SNPs and two functional SNPs (-1G>T and 518T>C) in FXR in 2355 Dutch IBD patients (1162 Crohn's disease and 1193 ulcerative colitis) and in 853 healthy controls. None of the SNPs was associated with inflammatory bowel disease, ulcerative colitis or Crohn's disease, nor with any clinical subgroup of Crohn's disease including CC [13]. Taken together, although all these mechanisms could have precluded FXR activation by CDCA or other FXR ligands in patients with Crohn's colitis, our current results clearly show that such activation is well feasible in this patient category.

Our findings provide a rationale to further explore the potential beneficial effects of FXR ligands in CC patients. Dysregulation of the immune response to intestinal bacteria is supposed to be a key mechanism in the pathogenesis of Crohn's disease, as illustrated by mutations in autophagy genes, NOD2 mutations and IL23 pathway mutations, with a resulting shift from secretion of anti-inflammatory mediators towards pro-inflammatory molecules. Activation of the Nuclear Factor kappa B (NF-κB) was identified as one of the key factors in this shift, resulting in strongly enhanced expression of pro-inflammatory genes, and recruitment of excess inflammatory cells to the intestinal wall. It has been shown that FXR activation can inhibit NF-κB in the intestine as well as in other organs [10], [12], [19] Phase 3 clinical trials are currently being performed to investigate the potential beneficial effects of a semisynthetic FXR ligand (6-ethyl-chenodeoxycholic acid) in chronic cholestatic liver diseases such as primary biliary cirrhosis. Of note, based on increase of plasma FGF19 levels, magnitude of FXR stimulation with CDCA 15 mg/kg in the current study appears similar to 10 mg 6-ethyl-chenodeoxycholic acid in primary biliary cirrhosis (the dose currently proposed in this cholestatic liver disease). (H.-U. Marschall, V. Luketic, A. Lövgren-Sandblom, L. Benthin, K. Kowdley et al. (2012).

Since FGF19 has been reported to induce gallbladder (GB) refilling in the mouse [3], we also determined concurrent plasma FGF19 levels and GB volumes during 6 hrs after the first CDCA ingestion and after 8 days CDCA. There were progressive increases with time for FGF19 levels and GB volumes, without any differences between CC and disease control groups. The initial decrease in plasma FGF 19 levels in a subset of CC patients and disease controls after first CDCA ingestion remains however unexplained. A similar phenomenon has been observed previously in normal subjects receiving an oral fat load [23] After the first CDCA dosage, there were no significant correlations between FGF19 levels and GB volumes at the individual time points. A potential explanation could be that initially, there is a strong increase of hepatic bile salt secretion and bile flow (and thus gallbladder filling), due to relatively large amounts of exogenous CDCA entering the enterohepatic circulation. As a result, effects of FGF19 on GB volumes may not be evident.

In summary, we found normal activation of the bile salt-ileal FXR axis in patients with CC using the endogenous FXR ligand chenodeoxycholic acid. These findings provide a rationale to further explore the potential therapeutic role of FXR agonists in this patient category.

## Supporting Information

Table S1 qRT-PCR primer list.
(DOC)

Checklist S1 CONSORT Checklist.
(DOC)

Protocol S1 Trial Protocol.
(DOC)
