# Supplementary material for: Pharmacological Activation of the Bile Acid Nuclear Farnesoid X Receptor Is Feasible in Patients with Quiescent Crohn's Colitis
Source: PLoS One. 2012 Nov 26;7(11):e49706. doi: 10.1371/journal.pone.0049706 (PMC3506649; doi:10.1371/journal.pone.0049706)
Supplement: Table S1 — qRT-PCR primer list. (DOC) [file pone.0049706.s001.doc]

| HRPT F | ATTGTAATGACCAGTCAACAGGG |
| --- | --- |
| HRPT R | GCATTGTTTTGCCAGTGTCAA |
| FXR F | CTACCAGGATTTCAGACTTTGGAC |
| FXR R | GAACATAGCTTCAACCGCAGAC |
| SHP F | AGGGACCATCCTCTTCAACC |
| SHP R | TTCACACAGCACCCAGTGAG |
| IBABP F | TCAGAGATCGTGGGTGACAA |
| IBABP R | TCACGCGCTCATAGGTCA |
| FGF19 F | CGTGCGGTACCTCTGCAT |
| FGF19 R | TCGGTACACATTGTAGCCATCT |
| iNOs F | TCGGCAGAATCTACAAAGTCC |
| iNOs R | TGGCCATCCTCACAGGAG |
| ANG1 F | CGTTTCTGCGGACTTGTTCT |
| ANG1 R | CGAAGACCAACAACAAAACG |
| ASBT F | TATTTCCTGTGGCGGGTTAC |
| ASBT R | GATGAGCGGGAAGGTGAATA |
