# Supplementary material for: Pharmacological Activation of the Bile Acid Nuclear Farnesoid X Receptor Is Feasible in Patients with Quiescent Crohn's Colitis
Source: PLoS One. 2012 Nov 26;7(11):e49706. doi: 10.1371/journal.pone.0049706 (PMC3506649; doi:10.1371/journal.pone.0049706)
Supplement: Protocol S1 — Trial Protocol. (DOC) [file pone.0049706.s003.doc]

**(Patho)Physiological aspects of the bile salt-FXR-FGF19 axis: potential consequences in Crohn’s disease**

University Medical Center Utrecht

Department of Gastroenterology and Hepatology

Heidelberglaan 100

3584 CX Utrecht

The Netherlands

**PHYSIOLOGICAL ASPECTS OF BILE SALT-FXR15/19 AXIS AND POTENTIAL CONSEQUENCES FOR CROHN’S DISEASE**

| **Protocol ID** | **NL27650.041.09** |
| --- | --- |
| **Short title** | **FXR-FGF19 functioning in IBD** |
| **Version** | **6** |
| **Date** | **25-11-10** |
| **Coordinating investigator** | **F.D.M. van Schaik, MD**  **University Medical Center Utrecht**  **Department of Gastroenterology and Hepatology**  **Huispostnummer F 02.618**  **Postbus 85500**  **3508 GA Utrecht**  **088-755 9811 / f.d.m.vanschaik@umcutrecht.nl** |
| **Principal investigator(s)** | **Dr. K.J. van Erpecum, MD, PhD**  **Department of Gastroenterology and Hepatology**  **088-7556270 / k.j.vanerpecum@umcutrecht.nl**  **Dr. B. Oldenburg, MD, PhD**  **Department of Gastroenterology and Hepatology**  **088-7557325 / b.oldenburg@umcutrecht.nl**  **Prof. dr. P.D. Siersema**  **Department of Gastroenterology and Hepatology**  **088-7559338 / p.d.siersema@umcutrecht.nl** |
| **Sponsor** | ***-*** |
| **Independent physician** | **Dr. F.P. Vleggaar, MD, PhD**  **University Medical Center Utrecht**  **Department of Gastroenterology and Hepatology**  **Huispostnummer F 02.618**  **Postbus 85500 3508 GA Utrecht**  **088-7553484 / f.p.vleggaar@umcutrecht.nl** |
| **Laboratory sites** | **Gastroenterology Research Laboratory, UMC Utrecht**  **Laboratory Clinical Chemistry**  **Heidelberglaan 100, Utrecht**  **AMC Liver Center**  **Meibergdreef 69-71, Amsterdam** |

**PROTOCOL SIGNATURE SHEET**

| **Name** | **Signature** | **Date** |
| --- | --- | --- |
| **Sponsor or legal representative:**  **Head of Department:**  **Prof. P.D. Siersema, MD, PhD**  **Department of Gastroenterology and Hepatology**  **088-7559338**  **p.d.siersema@umcutrecht.nl** |  |  |
| **Principal Investigator:**  **Dr. K.J. van Erpecum, MD, PhD**  **University Medical Center Utrecht**  **Department of Gastroenterology and Hepatology**  **Huispostnummer F 02.618**  **Postbus 85500**  **3508 GA Utrecht**  **088-7559338**  **k.j.vanerpecum@umcutrecht.nl** |  |  |

**TABLE OF CONTENTS**

1. INTRODUCTION AND RATIONALE [9](#__RefHeading___Toc206991995)

2. OBJECTIVES [10](#__RefHeading___Toc206991996)

3. STUDY DESIGN [11](#__RefHeading___Toc206991997)

4. STUDY POPULATION [14](#__RefHeading___Toc206991998)

4.1 Population (base) [14](#__RefHeading___Toc206991999)

4.2 Inclusion criteria [14](#__RefHeading___Toc206992000)

4.3 Exclusion criteria [15](#__RefHeading___Toc206992001)

4.4 Sample size calculation [16](#__RefHeading___Toc206992002)

5. TREATMENT OF SUBJECTS [17](#__RefHeading___Toc206992003)

5.1 Investigational product/treatment [17](#__RefHeading___Toc206992004)

5.2 Use of co-intervention (if applicable) [17](#__RefHeading___Toc206992005)

5.3 Escape medication (if applicable) [17](#__RefHeading___Toc206992006)

6. INVESTIGATIONAL MEDICINAL PRODUCT [18](#__RefHeading___Toc206992007)

6.1 Name and description of investigational medicinal product [18](#__RefHeading___Toc206992008)

6.2 Summary of findings from non-clinical studies [18](#__RefHeading___Toc206992009)

6.3 Summary of findings from clinical studies [18](#__RefHeading___Toc206992010)

6.4 Summary of known and potential risks and benefits [18](#__RefHeading___Toc206992011)

6.5 Description and justification of route of administration and dosage [18](#__RefHeading___Toc206992012)

6.6 Dosages, dosage modifications and method of administration [19](#__RefHeading___Toc206992013)

6.7 Preparation and labelling of Investigational Medicinal Product [19](#__RefHeading___Toc206992014)

6.8 Drug accountability [19](#__RefHeading___Toc206992015)

7. METHODS [20](#__RefHeading___Toc206992016)

7.1 Study parameters/endpoints [20](#__RefHeading___Toc206992017)

7.1.1 Main study parameter/endpoint [20](#__RefHeading___Toc206992018)

7.1.2 Secondary study parameters/endpoints (if applicable) [20](#__RefHeading___Toc206992019)

7.1.3 Other study parameters (if applicable) [20](#__RefHeading___Toc206992020)

7.2 Randomisation, blinding and treatment allocation [20](#__RefHeading___Toc206992021)

7.3 Study procedures [20](#__RefHeading___Toc206992022)

7.4 Withdrawal of individual subjects [21](#__RefHeading___Toc206992023)

7.4.1 Specific criteria for withdrawal (if applicable) [21](#__RefHeading___Toc206992024)

7.5 Replacement of individual subjects after withdrawal [21](#__RefHeading___Toc206992025)

7.6 Follow-up of subjects withdrawn from treatment [22](#__RefHeading___Toc206992026)

7.7 Premature termination of the study [22](#__RefHeading___Toc206992027)

8. SAFETY REPORTING [23](#__RefHeading___Toc206992028)

8.1 Section 10 WMO event [23](#__RefHeading___Toc206992029)

8.2 Adverse and serious adverse events 23

8.2.1 Suspected unexpected serious adverse reactions (SUSAR) [24](#__RefHeading___Toc206992031)

8.2.2 Annual safety report [24](#__RefHeading___Toc206992032)

8.3 Follow-up of adverse events [25](#__RefHeading___Toc206992033)

8.4 Monitoring 25

9. STATISTICAL ANALYSIS [26](#__RefHeading___Toc206992035)

9.1 Descriptive statistics [26](#__RefHeading___Toc206992036)

9.2 Univariate analysis [26](#__RefHeading___Toc206992037)

9.3 Multivariate analysis [26](#__RefHeading___Toc206992038)

9.4 Interim analysis (if applicable) [26](#__RefHeading___Toc206992039)

10. ETHICAL CONSIDERATIONS [27](#__RefHeading___Toc206992040)

10.1 Regulation statement [27](#__RefHeading___Toc206992041)

10.2 Recruitment and consent [27](#__RefHeading___Toc206992042)

10.3 Objection by minors or incapacitated subjects (if applicable) [27](#__RefHeading___Toc206992043)

10.4 Benefits and risks assessment, group relatedness [27](#__RefHeading___Toc206992044)

10.5 Compensation for injury [28](#__RefHeading___Toc206992045)

10.6 Incentives (if applicable) [28](#__RefHeading___Toc206992046)

11. ADMINISTRATIVE ASPECTS AND PUBLICATION [29](#__RefHeading___Toc206992047)

11.1 Handling and storage of data and documents [29](#__RefHeading___Toc206992048)

11.2 Amendments [29](#__RefHeading___Toc206992049)

11.3 Annual progress report [29](#__RefHeading___Toc206992050)

11.4 End of study report [29](#__RefHeading___Toc206992051)

11.5 Public disclosure and publication policy [30](#__RefHeading___Toc206992052)

12. REFERENCES [31](#__RefHeading___Toc206992053)

**LIST OF ABBREVIATIONS AND RELEVANT DEFINITIONS**

| **ABR** | **ABR form (General Assessment and Registration form) is the application form that is required for submission to the accredited Ethics Committee (ABR = Algemene Beoordeling en Registratie)** |
| --- | --- |
| **AE** | **Adverse Event** |
| **AR** | **Adverse Reaction** |
| **CA** | **Competent Authority** |
| **CCMO** | **Central Committee on Research Involving Human Subjects** |
| **CV** | **Curriculum Vitae** |
| **CDAI** | **Clinical Disease Activity Index** |
| **DSMB** | **Data Safety Monitoring Board** |
| **EU** | **European Union** |
| **EudraCT** | **European drug regulatory affairs Clinical Trials GCP Good Clinical Practice** |
| **IB** | **Investigator’s Brochure** |
| **IC** | **Informed Consent** |
| **IMP** | **Investigational Medicinal Product** |
| **IMPD** | **Investigational Medicinal Product Dossier** |
| **METC** | **Medical research ethics committee (MREC); in Dutch: medisch ethische toetsing commissie (METC)** |
| **(S)AE** | **Serious Adverse Event** |
| **SPC** | **Summary of Product Characteristics (in Dutch: officiële productinfomatie IB1-tekst)** |
| **Sponsor** | **The sponsor is the party that commissions the organisation or performance of the research, for example a pharmaceutical**  **company, academic hospital, scientific organisation or investigator. A party that provides funding for a study but does not commission it is not regarded as the sponsor, but referred to as a subsidising party.** |
| **SUSAR** | **Suspected Unexpected Serious Adverse Reaction** |
| **Wbp** | **Personal Data Protection Act (in Dutch: Wet Bescherming Persoonsgevens)** |
| **WMO** | **Medical Research Involving Human Subjects Act (Wet Medisch-wetenschappelijk Onderzoek met Mensen** |

**SUMMARY**

**Rationale:**

After a meal, gallbladder contraction evacuates bile salts into the intestine, with subsequent bile salt transport to the ileum and by active transport, reabsorption into the enterohepatic circulation. In the ileal enterocyte, reabsorbed bile salts activate the bile salt nuclear receptor FXR (Farnesoid X Receptor) with the result that: 1. toxic intracellular bile salt concentrations in the ileal enterocyte and in the liver cell are prevented by regulation of expression of various FXR target genes involved in intracellular bile salt transport and bile salt *neo*synthesis 2. “ileal brake” is activated through enhanced expression of the FXR target gene fibroblast growth factor (FGF) 19, which functions, after its secretion by the ileal cell, as a hormone inducing transition of post-prandial into fasting state, including gallbladder dilatation 3. adequate intestinal barrier function and antibacterial defense (both known to be disturbed in inflammatory bowel disease) are maintained, through regulation of expression of various pivotal FXR target genes.(1) In vitro studies suggest that an anti-inflammatory effect is generated through NFκB inhibition. Furthermore, preliminary evidence indicates that basal ileal FXR expression in patients with Crohn’s colitis is altered, suggesting a pathogenetic role for this nuclear receptor in Crohn’s disease. Indeed, in an animal model for colitis, synthetic FXR agonists ameliorated severity of colonic inflammation. (1)

**Objective**:

To study the functioning of the bile salt nuclear receptor FXR in patients with quiescent Crohn’s colitis.

**Study design:**

Prospective case control study.

**Study population:**

Twelve patients with quiescent Crohn’s colitis, defined as a Harvey-Bradshaw Index (HBI) score <= 4 scheduled for a surveillance colonoscopy. Twelve non-IBD patients scheduled for a colonoscopy to exclude pathology will serve as disease controls.

**Intervention**:

Fasting gallbladder volumes (as assessed by ultrasound) and plasma FGF19 concentration of both groups will be determined at baseline. Participants will receive the FXR agonist chenodeoxycholic acid (CDCA) 15 mg/kg for a period of 8 days. Fasting gallbladder volumes will be determined and blood samples for FGF19 analysis will be collected at baseline, every hour during the first 6 hours after CDCA ingestion and after 8 days of CDCA ingestion. Furthermore**,** one additional blood sample will be collected for future assessment of relevant SNPs at the day of the colonoscopy and fecal bile acid excretion will be determined after 24 hours of stool collection at the day before the colonoscopy. Ileal and cecal mRNA expression of FXR and relevant target genes will be determined after 8 days of CDCA ingestion. Differences in gallbladder, hormonal and fecal parameters between both groups will be compared.

**Main study parameters/endpoints:**

Primary study endpoint is the difference between Crohn’s patients and disease controls in increase in fasting plasma FGF19 concentration after 8 days CDCA ingestion.

Secondary study endpoints are the differences between Crohn’s patients and disease controls in: 1. acute increase of fasting plasma FGF19 concentration after CDCA ingestion: 2. increase of fasting gallbladder volumes after acute and 8 days CDCA ingestion; 3. expression in ileal and caecal biopsies of FXR and various target genes after CDCA ingestion; 4. fecal bile salt excretion after CDCA ingestion.

**Nature and extent of the burden and risks associated with participation, benefit and group relatedness:**

This is a mechanistic study and no direct benefits for patients in this study are expected. The risk of participation is considered to be very low. No relevant side effects have been noted during decades of experience with CDCA, except slightly softer stools and, exceptionally, mild increases of serum transaminases. (2-17) A total of 34 ml blood will be drawn from each patient. A hematoma may develop after venepuncture or placement of the cannula. Gallbladder volumes will be assessed by ultrasonography. Ultrasonographic examinations are non-invasive, safe procedures that will cause no harm to the patient. Stool collection will be performed during 24 hours. The colonoscopy is performed for clinical indications. These endoscopic examinations are routinely performed and are considered safe procedures. The risk associated with the additional biopsies taken for mRNA expression analysis is very low. (18)

# INTRODUCTION AND RATIONALE

*Neo*synthesis of primary bile salts (cholate and chenodeoxycholate) occurs in the liver from cholesterol. After conjugation to glycine or taurine, the bile salts are secreted at the apical side of the liver cell into the bile canalicular system and subsequently transported through the bile ducts and stored in the gallbladder, mainly as mixed micelles together with cholesterol and phosphatidylcholine. After meal ingestion, cholecystokinine release induces gallbladder contraction, with bile salt evacuation into the intestinal lumen (essential for adequate absorption of fat and fat-soluble vitamins). Bile salts are then transported to the ileum, and largely (>95%) reabsorbed through active transport by the ileal enterocyte into the entero-hepatic circulation. Recent data, mostly from animal experiments, indicate an important role for the bile salt-FXR-FGF15/19 axis in bile acid homeostasis: bile salts reabsorbed in the ileal cell activate the bile salt nuclear receptor FXR, with subsequent expression of various target genes (ASBT, ILBP, OST alpha and beta) to prevent excessive intracellular bile salt concentrations. Second, ileal FXR activation also induces secretion of the hormone FGF15 (human ortholog FGF19) by the ileal cell, with gallbladder dilatation and inhibition of hepatic bile salt *neo*synthesis. (19;20) It is hypothesized that FGF15 functions as “ileal brake”, signalling end of postprandial and return to interdigestive phase. Third, based on experiments in FXR “knock out” and wild type mice, FXR activation appears essential for antibacterial defense and to maintain intestinal barrier function. Specifically, putative FXR target genes as angiogenin 1, iNOS, CAR 12 and Il18 may be involved in maintaining adequate intestinal barrier function, normal intestinal permeability and prevention of intestinal bacterial overgrowth. (1) Fourth, based on *in vitro* data in our lab, FXR activation exerts anti-inflammatory effects, mediated through inhibition of NF-κB. From a pathophysiologic point of view, disordered function of bile salt-FXR-FGF15/19 axis is thought to be important in cholestatic liver diseases (i.e. inappropriately low FGF15/19, therefore no inhibition of hepatic bile salt neosynthesis, leading to high amounts of serum bile salts) and intestinal bacterial overgrowth.

Patients with Crohn’s colitis are characterized by impaired antibacterial defense and impaired intestinal barrier function, and according to recent preliminary data, altered FXR activation in the ileum. (21) In a mouse model for colitis, administration of a synthetic FXR agonist decreases severity of the inflammation. (1) In patients with Crohn’s disease, absorption of bile salts in the ileum into the enterohepatic circulation is thought to be impaired, either through active ileal inflammation or through faster passage of intestinal contents through small and large intestinal tract. We hypothesize that this may lead to impaired activation of intestinal FXR and FXR target genes involved in antibacterial defense. Also, constitutively decreased ileal FXR expression (for example due to polymorphisms in the FXR gene) could lead to less activation of the bile salt nuclear receptor in patients with Crohn’s colitis.

# OBJECTIVES

**Primary Objective:**

To study potential abnormal functioning of the bile salt-FXR axis in patients with clinically quiescent Crohn’s colitis. We will employ in this study the potent FXR ligand chenodeoxycholic acid to stimulate this axis and use ileal and cecal biopsies for quantification of activation of FXR and its target genes. Also, plasma FGF 19 concentration and gallbladder volume will be used as parameters for FXR activation.

# STUDY DESIGN

Patients with Crohn’s colitis with an indication for surveillance colonoscopy (according to the AGA guideline) and disease controls who need to undergo colonoscopy for clinical reasons to exclude significant disease are candidates for this study. Patients visiting the outpatient clinic and who meet the in- and exclusion criteria (4.2 and 4.3) will be asked for their interest to participate in this study by their treating physician. If patients are interested, patients will receive oral and written information about the study from the investigator and within 1-2 weeks they will be contacted by the investigator who will answer remaining questions and who will check the disease activity. If patients consent to participate in the study, they will be asked to visit the endoscopy department 7 days before the scheduled colonoscopy. During this visit, informed consent will be signed by both the patient and the investigator. When the signed informed consent is obtained, disease activity index scoring, fasting gallbladder volume determination by ultrasound (22) and collection of 3 ml blood from a peripheral venous cannula for determination of plasma FGF19 concentration will be performed. Thereafter, the FXR ligand chenodeoxycholic acid (CDCA) will be ingested (15 mg/kg), followed by ultrasonographic determination of gallbladder volume and collection of 3 mL blood every hour during 6 hours. The next six days CDCA (15 mg/kg) is ingested at bedtime. Patients will collect stools during 24 hours at the day preceding the colonoscopy. On the day of the clinically indicated colonoscopy patients are fasted, with the exception that ingestion of water is allowed. In the early morning, before colonoscopy, CDCA (15 mg/kg) is ingested. Upon arrival at the outpatient clinic fasting gallbladder volume is assessed by ultrasound and 3 ml of blood is drawn for plasma FGF19 determination. One additional blood sample (10 ml) will be collected for future assessment of relevant SNP’s. Thereafter, patients will receive the standard bowel preparation. During colonoscopy 6 biopsies in the ileum and cecum are taken. These will be immediately placed in liquid nitrogen and stored at -80°C for determination at a later stage of mRNA expression levels of FXR, Angiogenin 1, FGF19, iNOS, CAR12 and other FXR target genes of potential relevance. The schematic outline of the study is depicted in figures 1 and 2.

Fig. 1. Schematic outline of the study

| **Activity / Time (days)** | **X** | **14** | **-7** | **-6** | **-5** | **-4** | **-3** | **-2** | **-1** | **0** |
| --- | --- | --- | --- | --- | --- | --- | --- | --- | --- | --- |
| **Patient recruitment** | **x** |  |  |  |  |  |  |  |  |  |
| **Written information** | **x** |  |  |  |  |  |  |  |  |  |
| **Oral information (telephone)** |  | **x** |  |  |  |  |  |  |  |  |
| **Informed consent** |  |  | **x** |  |  |  |  |  |  |  |
| **Disease activity index** |  |  | **x** |  |  |  |  |  |  |  |
| **Body length / weight** |  |  | **x** |  |  |  |  |  |  |  |
| **Gallbladder volume(s)** |  |  | **x** |  |  |  |  |  |  | **x** |
| **Blood samples** |  |  | **x** |  |  |  |  |  |  | **x** |
| **CDCA ingestion** |  |  | **x** | **x** | **x** | **x** | **x** | **x** | **x** | **x** |
| **Stool collection** |  |  |  |  |  |  |  |  | **x** |  |
| **Bowel preparation** |  |  |  |  |  |  |  |  |  | **x** |
| **Drug accountability** |  |  |  |  |  |  |  |  |  | **x** |
| **Colonoscopy** |  |  |  |  |  |  |  |  |  | **x** |

Fig. 2. Study procedures


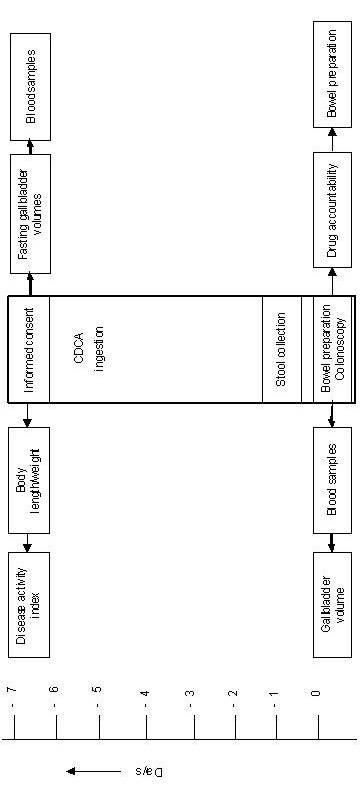


# STUDY POPULATION

## Population (base)

Twelve patients with clinically quiescent (HBI score <= 4) Crohn’s colitis scheduled for a surveillance colonoscopy and twelve disease controls scheduled for a colonoscopy to exclude significant disease will be enrolled in the study.

**24**

**Clinically indicated colonoscopies**

**12**

**Crohn’s disease**

**12**

**Disease controls**

Fig. 3. Study population

## Inclusion criteria

**Patients with Crohn’s disease:**

# Surveillance colonoscopy for established Crohn’s disease of the colon (indicated for clinical reasons)

# Informed consent of the patient.

**Disease** **controls:**

- A clinically indicated colonoscopy to exclude significant disease of the colon
- Informed consent of the patient

## Exclusion criteria

**Patients with Crohn’s disease:**

- HBI score > 4 or frequency of defaecation > 4 / day

# Serum C-reactive protein >20 (according to the last measurement, measured at most 3 months before the study)

# Surgery of the gastro-intestinal tract (only appendectomy is allowed)

# Previous cholecystectomy

# Gallbladder or bile duct stones

# Previous ERCP with papillotomy.

# Age < 18 years

# Inability to communicate with the patient

- Body Mass Index > 30
- Concomitant primary sclerosing cholangitis or other significant hepatic or biliary pathology
- Any malignancy within 5 years before the study
- Clotting disorders: prolonged prothrombin time (PT) > 2.5 seconds increased compared to control or activated partial thromboplastin time (APTT) > 9 seconds increased compared to control (these values are considered within the normal range) within 3 months before the study
- Use of steroids, cyclosporine, methotrexate, anti-TNF compounds, antibiotics or loperamide/codeine within one month before the study.
- Use of drugs, potentially interfering with CDCA (e.g. ursodeoxycholic acid or bile salt sequestrants), within one month before the study
- Pregnancy or lactation
- Liver function disorders: ASAT, ALAT, LDH, gGT and/or AF increased above ULN within 3 months before the study

**Disease controls:**

- Previous inflammation of the gastrointestinal tract (excluding previous infectious gastroenteritis if>6 months ago)
- Frequency of defaecation > 4 / day

# Serum C-reactive protein >20 (according to the last measurement, measured at most 3 months before the study)

# Surgery of the gastro-intestinal tract (only appendectomy is allowed)

# Previous cholecystectomy

# Gallbladder or bile duct stones

# Previous ERCP with papillotomy.

# Age < 18 years

# Inability to communicate with the patient

- Body Mass Index > 30
- Concomitant primary sclerosing cholangitis, or other significant hepatic or biliary pathology
- Any malignancy within 5 years before the study
- Clotting disorders: prolonged prothrombin time (PT) > 2.5 seconds increased compared to control or partial thromboplastin time (PTT) > 9 seconds compared to control (these values are considered normal) within 3 months before the study
- Use of steroids, cyclosporine, methotrexate, anti-TNF compounds, antibiotics or loperamide/codeine within one month before the study
- Use of drugs, potentially interfering with CDCA (e.g. ursodeoxycholic acid or bile salt sequestrants), within one month before the study
- Pregnancy or lactation
- Liver function disorders: ASAT, ALAT, LDH, gGT, and/or AF increased above ULN within 3 months before the study

## Sample size calculation

No data in human regarding the effect of chenodeoxycholic acid on plasma FGF19 concentration, gallbladder volume and expression of FXR target genes in patients with Crohn’s disease have been reported. A formal statistical power analysis has therefore not been performed. Two previous studies reported a mean fasting plasma FGF19 concentration between 190 and 250 pg/ml in healthy individuals. (20;23) One of these studies demonstrated an increase of 250% after ingestion of chenodeoxycholic acid in gallstone patients. Assuming that the CDCA-stimulated increase of plasma FGF19 in gallstone patients equals normal individuals and that the increase is reduced with 30% in patients with Crohn’s disease compared to normal subjects, 12 patients per group are required to yield a statistical power of 0.8 (α = 0.05, Standard Deviation = 75 pg/ml).

# TREATMENT OF SUBJECTS

## Investigational product/treatment

Chenodeoxycholic acid (15 mg/kg) will be administered daily for a period of eight days. The risk associated with chenodeoxycholic acid is considered to be very low. There is extensive experience with chenodeoxycholic acid from dissolution therapy of cholesterol gallstones during decades (2-17) and with the exception of slightly increased stool frequency and minor increases of liver biochemistry, which are reversible after cessation of chenodeoxycholic acid ingestion, no relevant side effects have been noted. This applies to ursodeoxycholic acid as well, the product of in vivo bacterial 7-beta epimerization of chenodeoxycholic acid, which is registered for the dissolution of cholesterol gallstones and the treatment of patients with primary biliary cirrhosis and primary sclerosing cholangitis.

## Use of co-intervention

Individuals participating in this study should not use bile acid therapy (e.g. ursodeoxycholic acid) or other drugs potentially interfering with CDCA. Patients with Crohn’s disease should not use steroids. Maintenance therapy, such as azathioprine or 6-mercaptopurine, can be continued.

## Escape medication

In case of the theoretical possibility that patients experience serious complaints following chenodeoxycholic acid ingestion, they will be withdrawn from the study. If indicated, medical treatment will be started (to the discretion of the treating physician).

# INVESTIGATIONAL MEDICINAL PRODUCT

## Name and description of investigational medicinal product

Chenodeoxycholic acid (CDCA) is an endogenous bile acid synthesized in the human liver that improves biliary dissolution of cholesterol thus preventing gallstone formation. CDCA is registered for cholesterol gallstone dissolution therapy in patients with a functioning gallbladder who have an increased surgical risk or are inoperable. During chenodeoxycholic acid therapy, the percentage of chenodeoxycholic acid in the endogenous bile acid pool increases from 40% at baseline to approximately 80% during treatment. (14;15;24;25)

## Summary of findings from non-clinical studies

CDCA is a natural bile acid and ligand of the farnesoid X receptor (FXR). In mice it has been demonstrated that stimulation of FXR by a synthetic ligand (GW4064) induces genes involved in enteroprotection and inhibits bacterial overgrowth. (1) In vivo treatment of colitis-induced mice with 6-ethyl chenodeoxycholic acid, a semi-synthetic FXR ligand, was shown to attenuate organ injury and to reduce the expression of inflammation-related cytokines. (21)

## Summary of findings from clinical studies

CDCA has not been investigated in Crohn’s disease. There is however extensive experience with chenodeoxycholic acid from dissolution therapy of cholesterol gallstones during decades. (2-17) CDCA reduces cholesterol synthesis in the liver by inhibition of the enzyme HMG-CoA-reductase. The biosynthesis of bile salts decreases to a lesser extent, resulting in lower cholesterol saturation in bile.

## Summary of known and potential risks and benefits

CDCA is safe and free of any serious adverse effects. With the exception of slightly increased stool frequency and in exceptional cases, minor increases of liver biochemistry and cholesterol, which are reversible after cessation of chenodeoxycholic acid ingestion, no relevant side effects have been noted with the use of CDCA. (2-17)

## Description and justification of route of administration and dosage

Since CDCA is effective in the ileum, is should be ingested orally. The normal CDCA dosage for cholesterol gallstone therapy is 15 mg/kg a day. CDCA is ingested in capsules of 250 mg.

## Dosages, dosage modifications and method of administration

CDCA will be administered in the registered dosage of 15 mg/kg using 250 mg capsules, as depicted in figure 3.

| **Body weight (kg)** | **Daily no. of capsules** | **Daily dose (mg)** | **Total no. of capsules needed** |
| --- | --- | --- | --- |
| 41 – 57 | 3 | 750 | 24 |
| 58 – 74 | 4 | 1000 | 32 |
| 75 – 90 | 5 | 1250 | 40 |
| 91 – 107 | 6 | 1500 | 48 |

Figure 4. Chenodeoxycholic acid dosage scheme.

## Preparation and labelling of Investigational Medicinal Product

Chenodeoxycholic acid is delivered by sigma-tau Arzneimittel GmbH.

## Drug accountability

- The trial medication will be collected and stored at our hospital pharmacy
- The study medication will be handed over to the patient at the hospital visit 8 days before the clinically indicated colonoscopy
- At the hospital visit for the colonoscopy patients will return remaining study medication and a pill count will be performed
- At the end of the study the trial medication will be destroyed by the pharmacy of UMC Utrecht.

# METHODS

## Study parameters/endpoints

### Main study parameter/endpoint

Primary study endpoint is the difference between Crohn’s patients and disease controls in increase of fasting plasma FGF19 concentration after 8 days CDCA ingestion.

### Secondary study parameters/endpoints (if applicable)

Secondary study endpoints are the differences between Crohn’s patients and disease controls in: 1. acute increase of fasting plasma FGF19 concentration after CDCA ingestion: 2. increase of fasting gallbladder volumes after acute and 8 days CDCA ingestion; 3. expression in ileal and caecal biopsies of FXR and various target genes after CDCA ingestion; 4. fecal bile salt excretion after CDCA ingestion.

### Other study parameters (if applicable)

The clinical activity will be scored 7 days before the colonoscopy. For Crohn’s patients, the Harvey-Bradshaw Index will be scored that incorporates the number of liquid stools, the intensity of abdominal pain, and general well being at the day preceding the assessment, the presence or absence of an abdominal mass and the presence of an extra-intestinal manifestation at the day of the assessment. A score <= 4 is considered to indicate clinical remission. For patients without Crohn’s disease, an adjusted index will be scored.

Furthermore, age, sex, duration of disease, endoscopic and histological disease extension, body length and weight and current medication use will be documented.

## Randomisation, blinding and treatment allocation

Not applicable

## Study procedures

Fasting gallbladder volumes and plasma FGF19 concentration will be determined eight times during this study by ultrasound (seven times during the hospital visit seven days before the colonoscopy and one time prior to the colonoscopy). A total of 34 ml blood will be collected during this study from an indwelling cannula and by venepuncture (day of colonoscopy). During a period of eight days participants will ingest CDCA (15 mg/kg). The day before the colonoscopy, patients will collect their stools during 24 hours using collection jars with enclosed operating manuals and bring the collected stools when they visit our department for colonoscopy. Patients will receive one conventional colonoscopy that is scheduled for clinical indications. Bowel preparation before the examination will be performed according to the standard procedure in our hospital. In addition to the biopsies taken for surveillance (when indicated, on average 30-40 biopsies), three ileal biopsies and three cecal biopsies will be taken for determination of mRNA expression levels of FXR target genes (see below).

**Fecal bile salt excretion**

Quantification of fecal bile salt excretion will be performed in our central diagnostic laboratory.

**FGF19 analysis**

Blood will be collected and plasma will be prepared. Plasma’s will be stored at -80 °C or -20 °C. Plasma’s will be transported to the AMC Liver Center for FGF19 analysis. A sandwich enzyme-linked immunosorbent assay (ELISA) kit will be used for the colorimetric detection of FGF19 in plasma. (20)

**Target gene expression analysis**

RNA from frozen biopsies will be isolated using a RNeasy kit (Qiagen, Hilden, Germany) according to the manufacturer’s instructions. Traces of genomic DNA that may copurify are removed by on-column DNase I digestion during RNA isolation. Concentration of RNA samples will be determined and the purity estimated by spectrophotometry. Expression of target genes (a.o. iNOS, IL18, CAR, angiogenin 1, FXR) will be measured using qRT-PCR.

## Withdrawal of individual subjects

Subjects can leave the study at any time for any reason if they wish to do so without any consequences for their further medical treatment. The investigator can decide to withdraw a subject from the study for urgent medical reasons.

### Specific criteria for withdrawal (if applicable)

Not applicable.

## Replacement of individual subjects after withdrawal

After withdrawal patients will be replaced.

## Follow-up of subjects withdrawn from treatment

Patients withdrawn from this study have continued follow-up by their treating physician just as the participating patients after completing this study.

## Premature termination of the study

Not applicable.

# SAFETY REPORTING

## Section 10 WMO event

In accordance to section 10, subsection 1, of the WMO, the investigator will inform the subjects and the reviewing accredited METC if anything occurs, on the basis of which it appears that the disadvantages of participation may be significantly greater than was foreseen in the research proposal. The study will be suspended pending further review by the accredited METC, except insofar as suspension would jeopardise the subjects’ health. The investigator will take care that all subjects are kept informed.

## Serious adverse events and serious adverse reactions

Adverse events (AEs) are defined as any undesirable experience occurring to a subject during a clinical trial, whether or not considered related to the investigational drug. All adverse events reported spontaneously by the subject or observed by the investiga­tor or his staff will be recorded in the case report form (CRF).

Adverse reactions (ARs) are defined as all untoward and unintended responses to an investigational medicinal product related to any dose administered.

A serious AE or serious AR is any untoward medical occurrence or effect that at any dose

- results in death;

- is life threatening (at the time of the event);
- requires hospitalisation or prolongation of existing inpatients’ hospitalisation;
- results in persistent or significant disability or incapacity;
- is a congenital anomaly or birth defect;
- is a new event of the trial likely to affect the safety of the subjects, such as an unexpected outcome of an adverse reaction, lack of efficacy of an IMP used for the treatment of a life threatening disease, major safety finding from a newly completed animal study, etc.

All SAEs will be evaluated and judged by the principal investigators on their relationship to the study-medication.

As documented in the Summary of Product Characteristics, there are currently no SARs reported for the use of chenodeoxycholic acid for the described indication.

### Suspected unexpected serious adverse reactions (SUSAR)

Unexpected adverse reactions are adverse reactions, of which the nature, or severity, is not consistent with the applicable Summary of Product Characteristics (SPC) of the study medication.

The sponsor will report expedited the following SUSARs to the METC:

- SUSARs that have arisen in the clinical trial that was assessed by the METC;
- SUSARs that have arisen in other clinical trial of the same sponsor and with the same medicinal product, and that could have consequences for the safety of the subjects involved in the clinical trial that was assessed by the METC.

The remaining SUSARs are recorded in an overview list (line-listing) that will be submitted once every half year to the METC. This line-listing provides an overview of all SUSARs from the study medicine, accompanied by a brief report highlighting the main points of concern.

The sponsor will report expedited all SUSARs to the competent authority, the Medicine Evaluation Board and the competent authorities in other Member States.

The expedited reporting will occur not later than 15 days after the sponsor has first knowledge of the adverse reactions. For fatal or life threatening cases the term will be maximal 7 days for a preliminary report with another 8 days for completion of the report.

### Annual safety report

In addition to the expedited reporting of SUSARs, the sponsor will submit, once a year throughout the clinical trial, a safety report to the accredited METC, competent authority, Medicine Evaluation Board and competent authorities of the concerned Member States.

This safety report consists of:

- a list of all suspected (unexpected or expected) serious adverse reactions, along with an aggregated summary table of all reported serious adverse reactions, ordered by organ system, per study;
- a report concerning the safety of the subjects, consisting of a complete safety analysis and an evaluation of the balance between the efficacy and the harmfulness of the medicine under investigation.

## Follow-up of adverse events

All adverse events will be followed until they have abated, or until a stable situation has been reached. Depending on the event, follow up may require additional tests or medical procedures as indicated, and/or referral to the general physician or a medical specialist.

## Monitoring

Monitoring will be performed by Julius Clinical Research (I. Sikking). A detailed monitoring plan will follow.

# STATISTICAL ANALYSIS

## Descriptive statistics

Not applicable.

## Univariate analysis

Plasma FGF19 concentration and gallbladder volume at various time points, faecal bile salt excretion and expression of various genes in ileal/caecal biopsies will be compared between the two groups (Crohn’s patients and disease controls) using unpaired T-tests or Mann-Whitney U tests as appropriate. Gallbladder volumes and FGF 19 concentration at various time points in the same patients will be compared by paired T-tests or Wilcoxon tests as appropriate. A two-sided P-value < 0.05 is considered statistically significant.

## Multivariate analysis

Not applicable.

## Interim analysis (if applicable)

Not applicable.

# ETHICAL CONSIDERATIONS

## Regulation statement

The study will be conducted according to the principles of the Declaration of Helsinki (59th WMA General Assembly, Seoul, October 2008) and in accordance with the Medical Research Involving Human Subjects Act (WMO).

## Recruitment and consent

Patients will be selected from individuals who visit the outpatient clinic of the Department of Gastroenterology at the University Medical Centre Utrecht and/or who are scheduled for a colonoscopy for clinical reasons.

Patients who meet the in- and exclusion criteria (4.2 and 4.3) will be asked for their interest to participate in this study by their treating physician. If patients are interested, patients will receive oral and written information about the study from the investigator and within 1-2 weeks they will be contacted by the investigator who will answer remaining questions and who will check the disease activity. If patients consent to participate in the study, they will be asked to visit the endoscopy department 7 days before the scheduled colonoscopy. During this visit, two copies of the informed consent will be signed by both the patient and the investigator. One copy will be handed over to the patient; the other will be stored in the patient’s study file. The general practitioner will be informed about the patient’s participation.

## Objection by minors or incapacitated subjects (if applicable)

Not applicable.

## Benefits and risks assessment, group relatedness

There are no direct benefits for the patients in this study. The risk of participation is considered to be very low. The risk associated with chenodeoxycholic acid is considered to be very low; with the exception of slightly increased stool frequency and minor increases of liver biochemistry, which are reversible after cessation of chenodeoxycholic acid ingestion, no relevant side effects have been noted. A total of 34 ml blood will be drawn from the patient. A hematoma may develop after venepuncture or placement of the cannula. Gallbladder volumes will be determined by ultrasonography. Ultrasonographic examinations are non-invasive, safe procedures and cause little harm to the patient. The colonoscopy is indicated for clinical reasons. Endoscopic examinations are routinely performed and considered as safe procedures. Taking additional biopsies will extend the duration of the colonoscopy with 2-4 minutes. Taking biopsies is associated with exceedingly low risk of secondary bleeding. (18) The risk associated with the additional biopsies taken for mRNA expression analysis is therefore at most to a minor degree increased. Patients will bring one additional visit to our hospital. The traveling costs for this additional visit will be restituted. Furthermore, patients will receive one-hundred-and-fifty (150) euro once only for the time they will spend during the additional visit in our hospital.

## Compensation for injury

The sponsor/investigator has a liability insurance which is in accordance with article 7, subsection 6 of the WMO.

The sponsor (also) has an insurance which is in accordance with the legal requirements in the Netherlands (Article 7 WMO and the Measure regarding Compulsory Insurance for Clinical Research in Humans of 23th June 2003). This insurance provides cover for damage to research subjects through injury or death caused by the study.

1. € 450.000,-- (i.e. four hundred and fifty thousand Euro) for death or injury for each subject who participates in the Research;
2. € 3.500.000,-- (i.e. three million five hundred thousand Euro) for death or injury for all subjects who participate in the Research;
3. € 5.000.000,-- (i.e. five million Euro) for the total damage incurred by the organisation for all damage disclosed by scientific research for the Sponsor as ‘verrichter’ in the meaning of said Act in each year of insurance coverage.

The insurance applies to the damage that becomes apparent during the study or within 4 years after the end of the study.

## Incentives

Not applicable.

# ADMINISTRATIVE ASPECTS AND PUBLICATION

## Handling and storage of data and documents

At the moment of inclusion into the study, an inclusion form will be filled in on which a study number is allocated to each patient according to the order of inclusion into one of the two study groups. The first Crohn’s patient will receive trial number CD-FXR-1, the second patient will receive number CD-FXR-2. Likewise, the first two disease controls will receive trial numbers N(on)CD-FXR-1 and NCD-FXR-2. During the study, only trial numbers will be used to identify patients. Only the coordinating investigator (F.D.M. van Schaik) will have access to the documents linking the trial number to the individual subject. All study-related data will be documented in a paper case report form (CRF). The CRFs will be stored in a locked room with limited access. CRFs will be processed in a computerized database. Only the coordinating investigator, F.D.M. van Schaik, will have access to this database. When data are changed or added, a new version (number, date) will be created with a detailed description of the changed data.

Biopsies will be stored in a locked room with limited access.

After the study has ended, all data and documents will be stored for 15 years. Human materials will be kept for 15 years.

## Amendments

Amendments are changes made to the research after a favourable opinion by the accredited METC has been given. All amendments will be notified to the METC that gave a favourable opinion.

## Annual progress report

The sponsor/investigator will submit a summary of the progress of the trial to the accredited METC once a year. Information will be provided on the date of inclusion of the first subject, numbers of subjects included and numbers of subjects that have completed the trial, serious adverse events/ serious adverse reactions, other problems, and amendments.

## End of study report

The investigator will notify the accredited METC of the end of the study within a period of 8 weeks. The end of the study is defined as the last patient’s last visit.

In case the study is ended prematurely, the investigator will notify the accredited METC, including the reasons for the premature termination.

 Within one year after the end of the study, the investigator/sponsor will submit a final study report with the results of the study, including any publications/abstracts of the study, to the accredited METC.

## Public disclosure and publication policy

This study will be performed at the Department of Gastroenterology and Hepatology at the University Medical Center Utrecht. Results of this study will be published in a scientific journal and will be part of the doctoral thesis of F.D.M. van Schaik.

# REFERENCES

(1) Inagaki T, Moschetta A, Lee YK, Peng L, Zhao G, Downes M, et al. Regulation of antibacterial defense in the small intestine by the nuclear bile acid receptor. Proc Natl Acad Sci U S A 2006 Mar 7;103(10):3920-5.

(2) Barbara L, Roda E, Roda A, Sama C, Festi D, Mazzella G, et al. The medical treatment of cholesterol gallstones: experience with chenodeoxycholic acid. Digestion 1976;14(3):209-19.

(3) Bateson MC, Ross PE, Murison J, Bouchier IA. Effect of prolonged feeding with chenodeoxycholic acid on bile in patients with and without gallstones. Gut 1977 Aug;18(8):599-605.

(4) Bell GD, Whitney B, Dowling RH. Gallstone dissolution in man using chenodeoxycholic acid. Lancet 1972 Dec 9;2(7789):1213-6.

(5) Danzinger RG, Hofmann AF, Schoenfield LJ, Thistle JL. Dissolution of cholesterol gallstones by chenodeoxycholic acid. N Engl J Med 1972 Jan 6;286(1):1-8.

(6) Fromm H, Holz-Slomczyk M, Zobl H, Schmidt E, Schmidt FW. Studies of liver function and structure in patients with gallstones before and during treatment with chenodeoxycholic acid. Acta Hepatogastroenterol (Stuttg) 1975 Dec;22(6):359-69.

(7) Maton PN, Iser JH, Reuben A, Saxton HM, Murphy GM, Dowling RH. Outcome of chenodeoxycholic acid (CDCA) treatment in 125 patients with radiolucent gallstones. Factors influencing efficacy, withdrawal, symptoms and side effects and post-dissolution recurrence. Medicine (Baltimore) 1982 Mar;61(2):86-97.

(8) Mok HY, Bell GD, Dowling RH. Effect of different doses of chenodeoxycholic acid on bile-lipid composition and on frequency of side-effects in patients with gallstones. Lancet 1974 Aug 3;2(7875):253-7.

(9) Pedersen L, Bremmelgaard A. Hepatic morphology and bile acid composition of bile and urine during chenodeoxycholic acid therapy for radiolucent gallstones. Scand J Gastroenterol 1976;11(4):385-9.

(10) Petroni ML, Jazrawi RP, Lanzini A, Zuin M, Pazzi P, Fracchia M, et al. Repeated bile acid therapy for the long-term management of cholesterol gallstones. J Hepatol 1996 Nov;25(5):719-24.

(11) Podda M, Zuin M, Battezzati PM, Ghezzi C, de FC, Dioguardi ML. Efficacy and safety of a combination of chenodeoxycholic acid and ursodeoxycholic acid for gallstone dissolution: a comparison with ursodeoxycholic acid alone. Gastroenterology 1989 Jan;96(1):222-9.

(12) Schoenfield LJ, Lachin JM. Chenodiol (chenodeoxycholic acid) for dissolution of gallstones: the National Cooperative Gallstone Study. A controlled trial of efficacy and safety. Ann Intern Med 1981 Sep;95(3):257-82.

(13) Toouli J, Jablonski P, Watts JM. Treatment of gallstones by chenodeoxycholic acid. Med J Aust 1980 May 17;1(10):478-9.

(14) LaRusso NF, Thistle JL. Effect of litholytic bile acids on cholesterol absorption in gallstone patients. Gastroenterology 1983 Feb;84(2):265-71.

(15) Leiss O, von BK, Streicher U, Strotkoetter H. Effect of three different dihydroxy bile acids on intestinal cholesterol absorption in normal volunteers. Gastroenterology 1984 Jul;87(1):144-9.

(16) Marks JW, Lan SP, Baum RA, Habig RL, Hanson RF, Hersh T, et al. Low-dose chenodiol to prevent gallstone recurrence after dissolution therapy. Ann Intern Med 1984 Mar;100(3):376-81.

(17) Ponchon T, Barkun AN, Pujol B, Mestas JL, Lambert R. Gallstone disappearance after extracorporeal lithotripsy and oral bile acid dissolution. Gastroenterology 1989 Aug;97(2):457-63.

(18) Arora G, Mannalithara A, Singh G, Gerson LB, Triadafilopoulos G. Risk of perforation from a colonoscopy in adults: a large population-based study. Gastrointest Endosc 2009 Mar;69(3 Suppl):654-64.

(19) Choi M, Moschetta A, Bookout AL, Peng L, Umetani M, Holmstrom SR, et al. Identification of a hormonal basis for gallbladder filling. Nat Med 2006 Nov;12(11):1253-5.

(20) Lundasen T, Galman C, Angelin B, Rudling M. Circulating intestinal fibroblast growth factor 19 has a pronounced diurnal variation and modulates hepatic bile acid synthesis in man. J Intern Med 2006 Dec;260(6):530-6.

(21) Fiorucci S, Renga B, Rizzo G, Mencarelli A, Disante M, Pruzanski M, et al. Essential counter-regulatory role of farnesoid-X-receptor (FXR) for intestinal innate immunity. Gastroenterology 130[4], A-87. 2006.
Ref Type: Abstract

(22) Everson GT, Braverman DZ, Johnson ML, Kern F, Jr. A critical evaluation of real-time ultrasonography for the study of gallbladder volume and contraction. Gastroenterology 1980 Jul;79(1):40-6.

(23) Schaap FG, Triantis V, van der Paardt M. Evidence for regulation of human FGF19 gene expression by ileal FXR. Hepatology 44[4], 591A. 2006.
Ref Type: Abstract

(24) Pooler PA, Duane WC. Effects of bile acid administration on bile acid synthesis and its circadian rhythm in man. Hepatology 1988 Sep;8(5):1140-6.

(25) Meier PB, Ansel HJ, Shafer RB, Duane WC. Efficacy of chenodeoxycholic acid and ursodeoxycholic acid for lowering cholesterol saturation index of gallbladder in patients with a sphincterotomy. Gastroenterology 1988 Dec;95(6):1595-600.
